# Clinical Impact of a Novel MicroRNA Chemo-Sensitivity Predictor in Gastrooesophageal Cancer

**DOI:** 10.1371/journal.pone.0148070

**Published:** 2016-02-17

**Authors:** Mette Winther, Steen Knudsen, Jesper Dahlgaard, Thomas Jensen, Anker Hansen, Peter Buhl Jensen, Trine Tramm, Jan Alsner, Marianne Nordsmark

**Affiliations:** 1 Dept. of Experimental Clinical Oncology, Aarhus University Hospital, Aarhus, Denmark; 2 Medical Prognosis Institute, Hoersholm, Denmark; 3 Centre for Health Promotion and Rehabilitation, Faculty of Health Sciences, VIA University College, Aarhus, Denmark; 4 Dept. of Pathology, Aarhus University Hospital, Aarhus, Denmark; 5 Dept. of Oncology, Aarhus University Hospital, Aarhus, Denmark; University of Connecticut Health Center, UNITED STATES

## Abstract

**Background:**

miRNAs might be potentially useful biomarkers for prediction of response to chemotherapeutic agents, radiotherapy and survival. The aim of this retrospective study was to validate miRNA response predictors in a cohort of patients with gastrooesophageal cancer in order to predict overall survival (OS) and disease-specific survival (DSS).

**Material and Methods:**

The study population encompassed 53 patients treated with curative intend for loco-regional gastrooesophageal cancer. miRNA expression was quantified from pre-therapeutic and diagnostic, formalin-fixed, paraffin embedded tumour specimens using Affymetrix GeneChip miRNA 1.0 Array. Based on growth inhibition of the NCI60 panel in the presence of cisplatin, epirubicine and capecitabine, a miRNA based response predictor was developed. The Cox proportional hazards model was applied to assess the correlations of the response predictor with OS and DSS.

**Results:**

A univariate analysis demonstrated a statistical significant improvement of OS for patients who had undergone surgical resection with prediction scores above the median prediction score (HR: 0.41 (95% CI: 0.17–0.96). Adjusting for surgery and stage, this predictor was identified to be independently associated with both OS (HR: 0.37 (95% CI: 0.16–0.87)) and DSS (HR: 0.32 (0.12–0.87)).

**Conclusion:**

The miRNA profile predictive for sensitivity to cisplatin, epirubicine and capecitabine was shown to be independently associated with OS and DSS in patients with gastrooesophageal cancer.

## Introduction

Chemo- and radiotherapy are essential therapeutic strategies for cancer treatment. However, due to intrinsic or acquired treatment resistance, chemo- and radiotherapy fail to eliminate all tumour cells [1]. In addition, responses to the same treatment are often contrasting in patients with seemingly identical cancers and for patients not responding to treatment, the time lost might be irrecoverably damaging.

Precision medicine is a promising approach for improvement of therapy response and survival rates, taking into account genetic variation in addition to variation in gene expression. Hence, identification of biomarkers predictive for response to chemotherapeutic agents and radiotherapy is of great clinical value. In clinical cancer research, microRNAs (miRNAs) are biomarkers with a significant potential due to both tissue-specificity and aberrant expression in tumour cells [2]. miRNAs are small, endogenous, non-coding RNAs that act as posttranscriptional regulators, targeting mRNA for degradation or translational repression [3]. They are involved in the regulation of numerous biological processes including tumour genesis, functioning as tumour suppressors or oncogenes [3,4].

In addition, miRNA expression profiling has been shown to be associated with tumour progression and response to therapy, suggesting their use as prognostic and predictive biomarkers as well as their important role in regulation of drug sensitivity [5,6]. Identification of miRNAs predictive for treatment response offers a highly promising opportunity to direct clinical decisions for the choice of treatment and improve outcome of disease.

In this retrospective study of patients with gastrooesophageal cancer, a novel bioinformatic approach has been applied. Based on growth inhibition of 60 human cancer cell lines (NCI60) subjected to cisplatin, epirubicine and capecitabine, in addition to, baseline miRNA expression of the 60 cell lines, a miRNA-based response predictor to the three chemotherapeutic agents was developed. The aim of this study was to validate the response predictor in patients with loco-regional gastrooesophageal cancer treated with curative intent in order to predict outcome in terms of overall survival and disease-specific survival.

## Materials and Methods

### Patients

The study population consisted of 53 patients diagnosed with loco-regional gastrooesophageal cancer (gastro-oesophageal junction cancer (GEJ) or gastric cancer (GC)) with available pre-therapeutic and diagnostic, formalin-fixed, paraffin embedded (FFPE) tumour specimens with sufficient material for miRNA microarray analysis (at least 400 ng total RNA). All patients were diagnosed with loco-regional gastrooesophageal cancer in the period 2009–2012 and treated with curative intent at Dept. of Oncology, Aarhus University Hospital, Denmark. Clinico-pathological parameters were obtained from medical records and pathology reports. Only TNM-stages were available from the medical records and, hence, patients were retrospectively staged by the authors according to the fifth-seventh editions of the AJCC/UICC staging guidelines. The study was approved by The Central Denmark Region Committees on Health Research Ethics and the Danish Data Protection Agency and was conducted in accordance with the Helsinki declaration. Dispensation from procurement of informed consent from patients in the study population was given by The Central Denmark Region Committees on Health Research Ethics as to the study would not induce any health risk or would be to any burden to participants. Patient information was anonymized and de-identified prior to analysis.

All patients had been treated with perioperative chemotherapy with cisplatin, epirubicine and capecitabine. Not all patients received full dose chemotherapy. A miRNA predictor profile, correlated to treatment sensitivity in NCI60 of the three chemotherapeutic agents, was developed. Baseline patient and tumour characteristics are listed in Table 1.

**Table 1:** Baseline patient and tumour characteristics.

Characterstics	N = 53
**Diagnosis—no. (%)**	
Gastro-esophageal junction cancer	46 (87)
Gastric cancer	7 (13)
**Histology—no. (%)**	
Adenocarcinomas	46 (87)
Other	7 (13)
**Sex—no. (%)**	
Male	43 (81)
Female	10 (19)
**Age—yr**	
Median (range)	63 (32–75)
**Stage—no. (%)**	
I (IA-B)	9 (17)
II (IIA-B)	23 (44)
III (IIIA-C)	19 (37)
IV	1 (1)
Unknown	1 (1)
**Evaluation CT-scans—no. (%)**	
Complete response	
Regression	30 (56)
Stable disease	17 (32)
Progressive disease	3 (6)
Unknown	3 (6)
**Pathological response—no. (%)**	N = 49
Complete response	
Non-complete response	49 (100)

### RNA isolation and quantification of miRNA

Total RNA was extracted from five consecutive slides of 12 μm FFPE pre-treatment tumour sections using the RecoverAll^™^ Total Nucleic Acid Isolation Kit for FFPE (Ambion, Inc. 2130 Woodward St. Austin, TX) according to the manufacturer protocol. Verification of invasive carcinoma in haematoxylin and eosin stained FFPE slides was carried out by an experienced pathologist (TT). The mean fraction of tumour area (defined as the area of invasive carcinoma) was estimated to 57% (range: 5–100). Quantification of total RNA was performed using a NanoDrop spectrophotometer (NanoDrop Technology, Wilmington, Del). miRNA was labeled using FlashTag^™^ HSR Biotin RNA Labeling Kit (Affymetrix, Santa Clara, CA) and hybridized to GeneChip miRNA version 1.0 microarrays (Affymetrix, Santa Clara, CA) according to manufactorer’s details. Arrays were washed and stained on an Affymetrix Fluidics Station 450X and scanned on an Affymetrix G7 scanner according to manufacturer’s instructions. Normalization of miRNA microarray data was performed in R using Robust Multi-array Average (RMA). All miRNAs were measurable in all 53 patient tumour samples.

### Predictor development based on in vitro assay

To evaluate the correlation between miRNA expression and drug sensitivity, growth inhibition (GI50) vectors of 60 cell lines subjected to cisplatin, epirubicine and capecitabine were downloaded from the DTP web site. Correlation between miRNA expression and drug sensitivity was calculated for each miRNA–drug combination. MicroRNAs with a correlation above 0.25 (positively correlated miRNAs) or below -0.25 (negatively correlated miRNAs) were retained for each treatment (S1 Table), and then combined in order to predict response to the combination treatment. Hence, a miRNA response profile was developed in which expression levels were correlated to the sensitivity of cisplatin, epirubicine and capecitabine.

### Prediction of chemo-sensitivity in clinical samples

The normalized expression of each miRNA in a response profile was used to predict sensitivity by turning the miRNA expression levels into a single prediction score. Hence, for each patient, sensitivity to the received treatment strategy was calculated as the difference between the average of positively correlated miRNAs and the average of negatively correlated miRNAs: Prediction score = mean(positively correlated miRNAs)–mean(negatively correlated miRNAs). Each miRNA in the profile was given equal weight. Next, the prediction score was normalized to a scale from 0 to 100 by a linear transformation of the prediction score of all patient samples; a score of zero meant least sensitive and a score of 100 meant most sensitive to the given treatment. For each patient, a score of predicted sensitivity to the received treatment strategy was calculated.

### Statistics

A Statistical analysis plan, with pre-specified success criteria, was performed before initiation of the study. The scores of treatment sensitivity for each patient was predicted before unblinding of the clinical data. The primary outcome was overall survival (OS), defined as the time interval from diagnosis of gastrooesophageal cancer to death from any cause or last follow-up (Aug. 11^th^ 2014) and the secondary outcome disease-specific survival, defined as time from diagnosis to death from or with gastroesoophageal cancer or last day of follow-up (Aug. 11^th^ 2014).

Survival analyses were carried out by dichotomizing the prediction scores using the median score as a cut off. Patients were predicted sensitive above cut off and resistant below or equal to cut off. Survival estimates were calculated according to the Kaplan-Meier method and compared using the univariate Cox proportional hazards model to assess associations between prediction scores and OS or DSS. The assumption of proportional hazards was verified with log-minus-log plots and Schoenfeld’s residuals. Multivariate analyses were performed to adjust for factors of presumed prognostic relevance. Hazard ratios (HR) for both uni- and multivariate analyses are presented at 3 years with 95% confidence interval (CI). All statistical analyses were performed using STATA, Version 12 (StataCorp, College Station, TX, USA).

## Results

### Outcome analysis

The median follow-up time for all patients was 23 months (range: 4–54 months) and the 1- year OS rate was 79% (95% CI: 66–88) and the 3-year OS rate 44% (95% CI: 30–58). At time of evaluation, 30 patients (57%) had died of which 24 patients had died of primary cancer. Radiographic response was observed in 30 patients (60%) among 50 patients with available treatment evaluation CT-scans. No patients obtained pathological complete response (defined as no evidence of vital residual tumour cells remaining in the resected specimen after neoadjuvant chemotherapy). Baseline patient and tumour characteristics are listed in Table 1.

### Association between the predicted sensitivity and survival

Using the NCI60 cell line panel, sensitivity towards cisplatin, epirubicine and capecitabine was correlated to the baseline miRNA expression of 1756 human miRNAs and other small nucleolar RNAs in the same cell lines. Both miRNAs being positively correlated and negatively correlated with each of the three chemotherapeutic agents were identified. In S1 Table, miRNAs used to predict response are listed. By use of diagnostic and pre-therapeutic FFPE tumour samples from the study population, miRNA expression levels were measured. In order to predict sensitivity, miRNA expression levels of the response profile was turned into one single prediction score on a scale from zero to 100 as described previously. A score of zero represented low sensitivity and a score of 100 high sensitivity.

**Fig 1:**
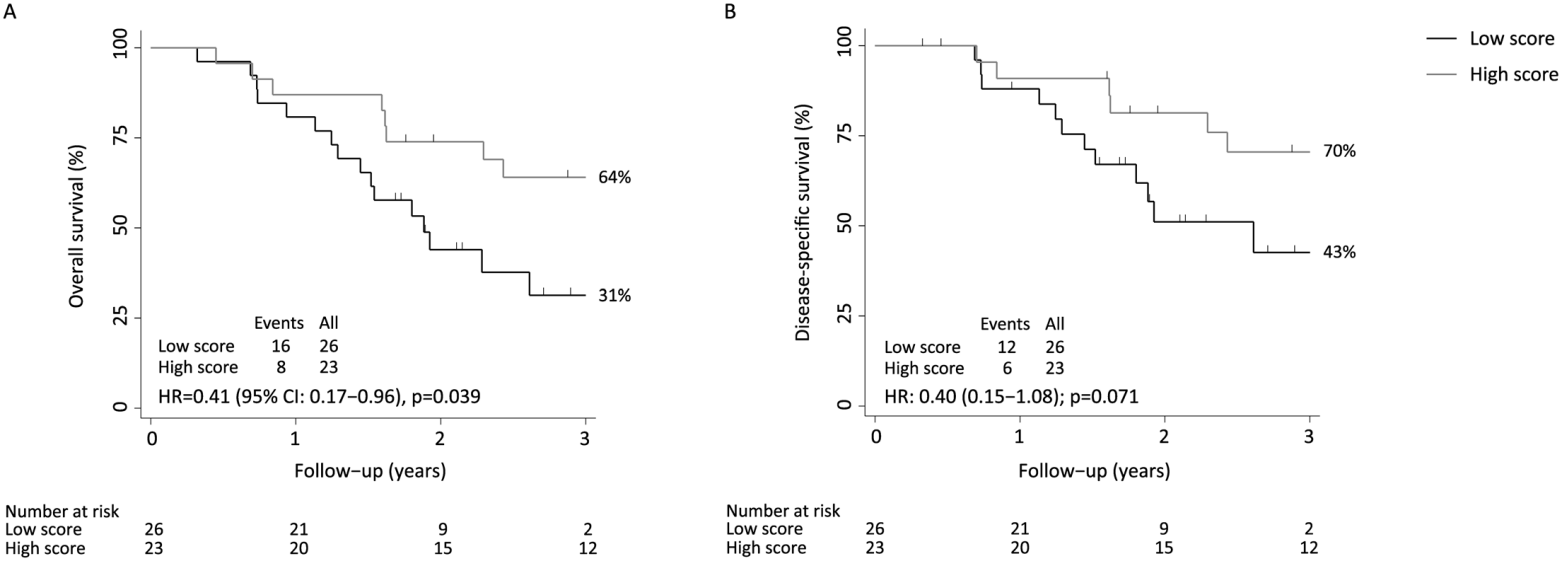
Kaplan-Meier estimates of (A) overall survival and (B) disease-specific survival of the combined prediction score of cisplatin, epirubicine, capecitabine for patients who had undergone surgical resection. Prediction scores are dichotomized by the median score.

When stratified by the median prediction score, trends towards increased OS (HR: 0.53 (95% CI: 0.25–1.14)) and DSS (HR: 0.57 (95% CI: 0.24–1.34)) were shown for patients with prediction scores above median. Surgery is the cornerstone of this treatment strategy and additional analysis of only those patients who had undergone surgical resection (N = 49) showed a significant improvement of OS (HR: 0.41 (95% CI: 0.17–0.96)) and a borderline significant enhanced DSS (HR: 0.40 (95% CI: 0.15–1.08)) for patients with prediction scores above median, Fig 1.

In a multivariate analysis, the prediction score was identified to be an independent prognostic marker for both OS (HR: 0.37 (95% CI: 0.16–0.87)) and DSS (HR: 0.32 (0.12–0.87)) after adjustment for surgery and stage, Table 2.

**Table 2:** Uni- and multivariate analyses.

	OS		DSS	
	HR (95% CI)	P	HR (95% CI)	P
**Univariate analysis**				
Age (>median age vs. < = median age)	1.10 (0.52–2.31)	0.810	0.87 (0.37–2.04)	0.749
Gender (male vs. female)	1.06 (0.40–2.80)	0.903	1.44 (0.42–4.86)	0.561
Histology (other vs. AC)	1.15 (0.40–3.30)	0.802	1.54 (0.52–4.57)	0.433
Surgery vs. non-surgery	0.10 (0.03–0.33)	<0.001	0.07 (0.02–0.24)	<0.001
Stage (III-IV vs. I-II)	1.85 (0.87–3.93)	0.112	2.01 (0.87–4.65)	0.102
Prediction score (>median vs. < = median)	0.53 (0.25–1.14)	0.103	0.57 (0.24–1.34)	0.195
**Multivariate analysis**				
Surgery vs. non-surgery	0.06 (0.02–0.24)	<0.001	0.04 (0.01–0.17)	<0.001
Stage (III-IV vs. I-II)	1.77 (0.82–3.83)	0.146	1.94 (0.82–4.55)	0.129
Prediction score (>median vs. < = median)	0.37 (0.16–0.87)	0.023	0.32 (0.12–0.87)	0.025

## Discussion

Gastrooesophageal cancers are malignancies with poor response rates to standard treatment regimens, resulting in a 5-year overall survival rate of only 15–25% [7,8]. miRNAs might be potentially useful biomarkers for prediction of response to chemotherapeutic agents. In this study, a miRNA profile predictive for sensitivity to cisplatin, epirubicine and capecitabine was developed in 53 patients with gastrooesophageal cancer. A univariate analysis including only patients who had undergone surgical resection showed a statistical significant improvement of OS for patients with prediction scores above cut off. Additionally, a multivariate analysis identified the prediction score as independently correlated with both OS and DSS. This promising result is encouraging and a new, larger prospective study is needed to validate the potential clinical utility of the predictor.

The development of the miRNA predictor profiles were based on a novel bioinformatic approach, previously published on diffuse large B-cell lymphoma (DLBCL) [9]. Using the NCI60 panel, two miRNA profiles were developed to which the expression levels correlated with sensitivity to the combination treatments of CHOP (cyclophosphamide, doxorubicin, vincristine and prednisone) and CHOEP (cyclophosphamide, doxorubicin, vincristine, etoposide and prednisone). In that study, the miRNA profiles successfully predicted response to treatment in patients with DLBCL.

Capecitabine is a prodrug that is enzymatically converted to 5-fluorouracil in the body. The *in vitro* derived response profiles for the two drugs have both overlaps and differences. In this study, we used the respective *in vitro* profile for the respective drug used in the clinic.

Prediction of response to combination therapy was performed by combining predictors for individual drugs. For the successful prediction of response, profiles for cisplatin, epirubicine and capecitabine were combined. When analysing the individual predictors in a multivariate analysis, the cisplatin and epirubicine predictors contributed most, while the capecitabine predictor contributed least (data not shown).

Therapy failure is widely observed in the clinical setting due to either intrinsic or acquired resistance to chemo- or radiotherapy. miRNAs have been shown to play important roles in the regulation of drug and radiation sensitivity in gastrooesophageal cancer. In oesophageal cancer, Hamano et al reported that overexpression of miR-200c was significantly correlated with poor response to cisplatin-based chemotherapy, potentially through up-regulation of the Akt-pathway (PPP2R1B) [10] and Tanaka et al showed that high pre-treatment expression levels of miR-200c in serum were significantly associated with impaired response to cisplatin-based chemotherapy [11]. In gastric cancer cells, however, overexpression of miR-200c has been correlated with reduced tumour growth and migration [12], in addition to enhanced cisplatin sensitivity [13]. These conflicting results indicate the various implications of miR-200c in different cancer types. In this study, miR-200c was included as predictor for response to epirubicine (negatively correlated).

In common with the miRNA response profile of the present study, several other studies have identified miRNAs to be associated with chemotherapeutic efficacy in gastrooesophageal cancer. For instance, hsa-let-7c has been shown to be associated with prognosis and to be a marker of sensitivity to cisplatin through the regulation of the IL-6/STAT3 pathway in patients with oesophageal cancer [14] and miR-141 has been found to exert anti-apoptotic effects that confers cisplatin resistance in oesophageal cancer cell lines, possibly through downregulation of YAP1 [15]. In the present study, has-let-7c was negatively correlated with capecitabine and miR-141 was negatively correlated with epirubicine. In another study, pre-treatment miR-505-star and miR-99b were shown to be significantly correlated with pathological response in patients with oesophageal adenocarcinoma [16]. In the present study, miR-505-star was negatively correlated with capecitabine and miR-99b negatively correlated with capecitabine and epirubicine.

In conclusion, the miRNA profile predictive for sensitivity to cisplatin, epirubicine and capecitabine was shown to be independently associated with OS and DSS in patients with gastrooesophageal cancer. This promising result is encouraging and a new, larger prospective study is warranted in order to validate the potential clinical utility of the predictor.

## Supporting Information

S1 Table Lists of microRNAs for all drug and combination predictors used. For each treatment the list of Affymetrix probesets on the miRNA version 1.0 array are shown with their Affymetrix ID. Probesets shown in roman are positively correlated, probesets shown in italics are negatively correlated. For combination treatments, individual profiles are combined.
(DOCX)
