# Supplementary material for: Clinical Impact of a Novel MicroRNA Chemo-Sensitivity Predictor in Gastrooesophageal Cancer
Source: PLoS One. 2016 Feb 17;11(2):e0148070. doi: 10.1371/journal.pone.0148070 (PMC4757421; doi:10.1371/journal.pone.0148070)
Supplement: S1 Table — For each treatment the list of Affymetrix probesets on the miRNA version 1.0 array are shown with their Affymetrix ID. Probesets shown in roman are positively correlated, probesets shown in italics are negatively correlated. For combination treatments, individual profiles are combined. (DOCX) [file pone.0148070.s001.docx]

**Table S1. Lists of microRNAs for all drug and combination predictors used.** For each treatment the list of Affymetrix probesets on the miRNA version 1.0 array are shown with their Affymetrix ID. Probesets shown in roman are positively correlated, probesets shown in italics are negatively correlated. For combination treatments, individual profiles are combined.

| **Treatment** | **List of microRNAs used to predict response** |
| --- | --- |
| cisplatin | ENSG00000202498_x_st HBII-436_st HBII-438A_s_st HBII-85-11_st HBII-85-26_st HBII-85-2_x_st HBII-85-6_x_st HBII-85-8_x_st U38A_st U43_st U43_x_st U48_st U55_st U55_x_st U57_st U74_x_st U95_st hsa-miR-124_st hsa-miR-1281_st hsa-miR-181a-star_st hsa-miR-181b_st hsa-miR-1825_st hsa-miR-342-3p_st hsa-miR-374b-star_st hsa-miR-410_st hsa-miR-432_st hsa-miR-433_st hsa-miR-485-3p_st hsa-miR-542-5p_st hsa-miR-631_st hsa-miR-654-3p_st hsa-miR-766_st hsa-miR-923_st  *hsa-miR-10a_st hsa-miR-148b_st hsa-miR-151-3p_st hsa-miR-151-5p_st hsa-miR-183_st hsa-miR-192-star_st hsa-miR-192_st hsa-miR-193b_st hsa-miR-194_st hsa-miR-200a-star_st hsa-miR-200a_st hsa-miR-200b-star_st hsa-miR-200b_st hsa-miR-203_st hsa-miR-29b_st hsa-miR-30b_st hsa-miR-30d_st hsa-miR-320d_st hsa-miR-429_st hsa-miR-449a_st hsa-miR-449b_st hsa-miR-552_st hsa-miR-584_st hsa-miR-625_st hsa-miR-7_st* |
| capecitabine | ACA10_s_st ACA18_x_st ACA44_st ACA48_x_st ACA55_st ACA61_st ACA9_st ENSG00000199411_s_st ENSG00000201859_x_st ENSG00000207002_st ENSG00000207002_x_st ENSG00000207098_st ENSG00000212604_st HBII-142_st HBII-142_x_st HBII-166_st HBII-336_st HBII-382_s_st HBII-55_st U15A_st U17b_st U22_st U3-2_s_st U36C_st U38B_st U38B_x_st U49A_st U51_st U55_x_st U56_st U71c_x_st U71d_st U71d_x_st U74_x_st U78_s_st U78_x_st U83B_st U95_st U96a_x_st U97_st U99_st hcmv-miR-UL70-3p_st hsa-let-7b-star_st hsa-miR-1183_st hsa-miR-1201_st hsa-miR-1203_st hsa-miR-1207-5p_st hsa-miR-1225-5p_st hsa-miR-1228-star_st hsa-miR-1228_st hsa-miR-1246_st hsa-miR-1249_st hsa-miR-1260_st hsa-miR-1268_st hsa-miR-1281_st hsa-miR-1298_st hsa-miR-1299_st hsa-miR-1300_st hsa-miR-1322_st hsa-miR-135b-star_st hsa-miR-145_st hsa-miR-149-star_st hsa-miR-150_st hsa-miR-15a-star_st hsa-miR-17-star_st hsa-miR-1826_st hsa-miR-18a-star_st hsa-miR-18a_st hsa-miR-195-star_st hsa-miR-202_st hsa-miR-297_st hsa-miR-302d_st hsa-miR-330-3p_st hsa-miR-335-star_st hsa-miR-338-5p_st hsa-miR-346_st hsa-miR-34b_st hsa-miR-486-3p_st hsa-miR-486-5p_st hsa-miR-491-3p_st hsa-miR-548c-5p_st hsa-miR-551b_st hsa-miR-572_st hsa-miR-574-5p_st hsa-miR-593-star_st hsa-miR-595_st hsa-miR-610_st hsa-miR-629-star_st hsa-miR-637_st hsa-miR-638_st hsa-miR-640_st hsa-miR-647_st hsa-miR-663b_st hsa-miR-768-3p_st hsa-miR-885-5p_st hsa-miR-886-3p_st hsa-miR-923_st hsa-miR-92a_st hsa-miR-92b-star_st hsa-miR-936_st hsa-miR-943_*st*  *hsa-let-7a_st hsa-let-7b_st hsa-let-7c_st hsa-let-7e-star_st hsa-let-7e_st hsa-let-7f_st hsa-miR-100_st hsa-miR-125a-3p_st hsa-miR-125a-5p_st hsa-miR-125b_st hsa-miR-1287_st hsa-miR-1301_st hsa-miR-130a_st hsa-miR-149_st hsa-miR-151-3p_st hsa-miR-151-5p_st hsa-miR-155_st hsa-miR-181d_st hsa-miR-193a-5p_st hsa-miR-193b-star_st hsa-miR-193b_st hsa-miR-196b_st hsa-miR-221-star_st hsa-miR-26a_st hsa-miR-29a-star_st hsa-miR-30a_st hsa-miR-30b-star_st hsa-miR-31-star_st hsa-miR-31_st hsa-miR-320d_st hsa-miR-335_st hsa-miR-505-star_st hsa-miR-542-5p_st hsa-miR-658_st hsa-miR-935_st hsa-miR-98_st hsa-miR-99a_st hsa-miR-99b-star_st hsa-miR-99b_st* |
| epirubicine | HBII-85-6_x_st U104_st U17b_st U17b_x_st U52_st U55_st U55_x_st hsa-miR-106a-star_st hsa-miR-106b-star_st hsa-miR-1183_st hsa-miR-124_st hsa-miR-127-3p_st hsa-miR-1281_st hsa-miR-1299_st hsa-miR-140-3p_st hsa-miR-153_st hsa-miR-154_st hsa-miR-181c_st hsa-miR-195-star_st hsa-miR-199a-3p_st hsa-miR-199a-5p_st hsa-miR-199b-3p_st hsa-miR-297_st hsa-miR-29b-2-star_st hsa-miR-33b-star_st hsa-miR-342-3p_st hsa-miR-346_st hsa-miR-34b_st hsa-miR-409-5p_st hsa-miR-431_st hsa-miR-432_st hsa-miR-433_st hsa-miR-629-star_st hsa-miR-631_st hsa-miR-654-3p_st hsa-miR-671-5p_st hsa-miR-766_st hsa-miR-92b_st  *hsa-let-7e_st hsa-miR-10a-star_st hsa-miR-10a_st hsa-miR-10b_st hsa-miR-125a-3p_st hsa-miR-125a-5p_st hsa-miR-141-star_st hsa-miR-141_st hsa-miR-151-3p_st hsa-miR-151-5p_st hsa-miR-192_st hsa-miR-193b_st hsa-miR-194_st hsa-miR-200a-star_st hsa-miR-200a_st hsa-miR-200b-star_st hsa-miR-200b_st hsa-miR-200c-star_st hsa-miR-200c_st hsa-miR-203_st hsa-miR-221-star_st hsa-miR-23a_st hsa-miR-29b-1-star_st hsa-miR-29b_st hsa-miR-30a-star_st hsa-miR-30a_st hsa-miR-30b-star_st hsa-miR-30c-2-star_st hsa-miR-30c_st hsa-miR-30d_st hsa-miR-30e-star_st hsa-miR-371-3p_st hsa-miR-371-5p_st hsa-miR-372_st hsa-miR-373_st hsa-miR-429_st hsa-miR-516a-5p_st hsa-miR-720_st hsa-miR-934_st hsa-miR-99b-star_st hsa-miR-99b_st* |
|  |  |
